# Biological Features of Bone Marrow Mesenchymal Stromal Cells in Childhood Acute Lymphoblastic Leukemia

**DOI:** 10.4274/tjh.2017.0209

**Published:** 2018-03-06

**Authors:** Stella Genitsari, Eftichia Stiakaki, Chryssoula Perdikogianni, Georgia Martimianaki, Iordanis Pelagiadis, Margarita Pesmatzoglou, Maria Kalmanti, Helen Dimitriou

**Affiliations:** 1 Crete University Faculty of Medicine, University Hospital of Heraklion, Department of Pediatric Hematology and Oncology, Crete, Greece; 2 Crete University Faculty of Medicine, Department of Pediatrics, Crete, Greece; 3 Crete University Faculty of Medicine, Division of Mother and Child Health, Crete, Greece; 4 Metropolitan Hospital, N. Faliro, Athens, Greece; 5 Private Sector

**Keywords:** Bone marrow microenvironment, Childhood leukemia, Mesenchymal stromal cells, Stromal cell-derived factor 1α

## Abstract

**Objective:**

Mesenchymal stromal cells (MSCs) have a supportive role in hematopoiesis and as components of the bone marrow (BM) microenvironment may present alterations during acute lymphoblastic leukemia (ALL) and be affected by chemotherapeutic agents. We examined the biological and functional characteristics of MSCs in ALL diagnosis and treatment and their effect on MSC qualitative properties.

**Materials and Methods:**

Immunophenotypic characterization, evaluation of clonogenicity, and proliferative capacity were measured. Apoptotic features, cell-cycle analysis, and stromal cell-derived factor 1α and angiopoietin-1 levels in MSC supernatant at diagnosis and in different phases of treatment were assessed. Chemotherapy was administered according to the Berlin-Frankfurt-Munster-2000 protocol. BM samples from children with solid tumors without BM involvement were used as the control group.

**Results:**

The morphology, the immunophenotypic profile, and the apoptotic characteristics of the MSCs were not affected by leukemia. The secretion of factors involved in the trafficking of hematopoietic cells in the BM seems to be upregulated at diagnosis in comparison to the treatment phases. MSCs are influenced by the disease in terms of their functional characteristics such as clonogenicity and proliferation rate. These effects cease as soon as treatment is initiated. Chemotherapy does not seem to exert any effect on any of the MSC features examined.

**Conclusion:**

MSCs from children with ALL are affected by their interaction with the leukemic environment, but this phenomenon ceases upon treatment initiation, while no effect is observed by chemotherapy itself.

## Abstract (tr)

**Amaç:**

Mezenkimal stroma hücreleri (MSH) hematopoezde destek rolü oynar, kemik iliği (Kİ) mikroçevresinin parçası olduklarından akut lenfoblastik lösemide (ALL) değişikliğe uğrayabilir ve kemoterapötik ajanlardan etkilenebilirler. Bu çalışmada, ALL’de tanı anında ve tedavide MSH’lerin biyolojik ve fonksiyonel özellikleri ile bunların MSH’lerin niteliksel özellikleri üzerine olan etkilerini araştırdık.

**Gereç ve Yöntemler:**

İmmünofenotipik özellikler, klonalite değerlendirilmesi ve çoğalma kapasitesi ölçümleri yapıldı. Tanıda ve tedavinin değişik evrelerinde MSH süpernatanında apoptotik özellikler, hücre döngüsü analizi ve stromal hücre türevi factor-1α ile anjiyopoietin-1 düzeyleri değerlendirildi. Kemoterapi olarak Berlin-Frankfurt-Munster-2000 protokolü uygulandı. Solid tümörü olan ve Kİ tutulumu bulunmayan hastaların Kİ örnekleri kontrol grubu olarak kullanıldı.

**Bulgular:**

MSH’lerin morfoloji, immünofenotipik profil ve apoptotik özellikleri açısından lösemiden etkilenmediği görüldü. Hematopoetik hücrelerinin Kİ’de yer değiştirmesi üzerine etkisi olabilen faktörlerinin salınımının tanıda, tedavi evrelerine göre upregüle olduğu tespit edildi. MSH’ler hastalıktan klonalite ve çoğalma hızı gibi fonksiyonel özellikler kapsamında etkilenmekteydi. Bu etkiler tedavi başlanması ile duraklamaktaydı. Kemoterapinin incelenen MSH özelliklerinden hiçbiri üzerine bir etkisi olmadığı görüldü.

**Sonuç:**

ALL’si olan çocuklardaki MSH’ler lösemik çevre ile ilişkilerden etkilenir, ancak bu fenomen tedavi başlanması ile duraklar ve bu çalışmada kemoterapinin bunun üzerine bir etkisi gözlenmemiştir.

## Introduction

Mesenchymal stromal cells (MSCs) constitute part of the bone marrow (BM) microenvironment where the survival, proliferation, and differentiation of hematopoietic stem cells (HSCs) take place [1]. Despite the large amount of information on the nature of MSCs, they have not been fully characterized so far. The in vivocounterparts or possibly precursors of culture-developed MSCs are currently considered to be perivascular cells, namely pericytes. These two-cell populations share similar properties in terms of marker expression, ability to self-renew, and potential to differentiate into multiple cell types such as adipocytes, chondrocytes, osteocytes, and myocytes under specified culture conditions [2,3]. The BM microenvironment is believed to play a pivotal role in the development and progression of leukemia [4]; thus, it is reasonable to speculate that MSCs may also be involved in the perturbation of normal hematopoiesis. Their putative role in oncogenesis and leukemogenesis has not been fully clarified and the results from the studies already published are contradictory. In vitro studies have shown that MSCs from newly diagnosed adult patients with leukemia (acute myeloid leukemia and acute lymphoblastic leukemia) are less efficient for supporting normal hematopoietic progenitor cell survival and this functional capacity is partially restored after chemotherapy [5]. Their implication in childhood ALL has only recently being addressed, revealing that ALL-MSCs display reduced proliferative capacity and ability to support long-term hematopoiesis in vitro while those isolated at diagnosis did not differ from those obtained during treatment [6]. The detection of leukemia-associated genetic aberrations in MSCs implied a clonal relationship between MSCs and leukemia cells in childhood ALL and suggested the involvement of MSCs in the pathogenesis of the disease [7]. Involvement of MSCs in various malignancies via deregulation of the secretion of chemokines [8,9,10] implies that they mediate cell migration and homing [11]. Stromal cell-derived factor 1a (SDF-1α or CXCL12) was found to retain and support the HSCs in the BM via the SDF-1α/CXCR4 axis [12,13]. CXCL12 is constitutively secreted by marrow stromal cells, being the major source for CXCL12 in adults [14]. Less is known about its role in hematological malignancies and how it could be affected during chemotherapy. The existing studies have come to conflicting results [8,15]. Angiopoietin-1 (Ang-1), initially known for its role in both embryonic and postnatal angiogenesis, has recently been reported to interact with HSC-expressed Tie-2 [3,16], enhancing the maintenance of HSCs in a quiescent state within the BM, and Ang-1 is thereby part of the network regulating the “stemness” of HSCs [17].

MSCs have been considered promising candidates for cell therapies and, in view of their potential, there are many ongoing studies to understand their properties, mechanisms of action, and putative role in hematological malignancies [7,18,19,20]. So far MSCs from different sources have been shown to exhibit different properties [21]. Moreover, BM MSCs from children seem to be different from their adult counterparts [22].

The aim of this study is to characterize MSCs derived from the BM of children with ALL at the onset of the disease in order to evaluate the leukemic effect, if any, on their biological/functional properties. In addition, an attempt was made to compare this population with the MSCs derived from the BM during different treatment phases for the assessment of the effect of chemotherapy on these features.

## Materials and Methods

### Patients

BM samples from children with B-lineage ALL and >90% BM infiltration at diagnosis, hospitalized from 2006 to 2010 at the Department of Pediatric Hematology and Oncology, University Hospital of Heraklion, were studied. They included samples at diagnosis (d, n=28), day 15 (d15, n=12), day 33 of induction therapy (d33, n=20) when remission was achieved, at intensification-consolidation (consol, n=33), during maintenance (maint, n=19) therapy, and at the end of treatment (end, n=20), all in remission. MSCs examined at different phases of ALL treatment are not necessarily in all cases from the same patients. Patients were treated according to the ALL Berlin-Frankfurt-Munster-2000 protocol and their risk stratification [medium risk (MR) and high risk (HR)] according to the same protocol was considered in some of the employed assays. The control group (n=15) consisted of BM samples from children with solid tumors without BM involvement. Patients’ ages ranged from 1.2 to 18 years (median: 6 years). The study was approved by the Ethical Committee of the University Hospital of Heraklion.

Methods are described in more detail in the Appendix (Supplementary Materials and Methods).

### BM Mononuclear Cells (MNCs) Isolation and MSC Culture and Expansion

BM MNCs, following Ficoll-Hypaque separation (1077 g/mL; Lymphoprep, Nycomed, Oslo, Norway), were cultured in a-MEM as described previously for MSC development [22]. MSCs were maintained for up to five passages. Assays were performed at any of P1 to P4 depending on the cell availability.

### Immunophenotyping Evaluation

Phenotypic characterization of MSCs was performed by flow cytometry at various passages using hematopoietic cell and MSC-specific monoclonal antibodies (BD Biosciences, San Jose, CA, USA). One hundred thousand cells were stained with the markers as described previously [23]. At least 10,000 events were acquired for each analysis.

### Cell Doubling Time (DT)

DT was calculated according to the formula DT=t/*n*=t×log(2)/log (cells harvested/cells inoculated), where t is the time between initial plating and harvest for the respective passage.

### Colony Forming Units-Fibroblast (CFU-F) Formation

At day 0, 1x10^5^ MNCs were seeded in each well of a 24-well plate (in triplicate) in the absence of fibroblast growth factor-2 (FGF-2). At subsequent passages, MSCs were plated in 20-cm^2^ petri plates at a concentration of 10 cells/cm^2^ (in duplicate). The colonies that developed were categorized according to their size as small (S), medium (M), and large (L, highly proliferating) CFU-F. The sum of all sizes is denoted as CFU-F.

### Cell-Cycle Analysis - Apoptosis

MSCs at either P2 or P3 were stained with propidium iodide in order to estimate the percentage of cells in each phase of the cell cycle. Cell-cycle analysis was performed using WinMDI software version 2.8 [24].

Apoptotic MSCs at passages P2 and P4 were detected by flow cytometry and 7-amino-actinomycin D (7-AAD; Sigma, St. Louis, MO, USA) staining [25].

### Detection of SDF-1α and Ang-1 (ELISA)

A quantitative sandwich enzyme-linked immunosorbent assay technique (ELISA) was employed for the determination of both SDF-1α and Ang-1 (R&D Systems, Minneapolis, MN, USA) in the supernatant of MSCs at any of P1 to P3 cultures (and of MNCs at d0) within the leukemia group only, at diagnosis, and during treatment phases following the instructions of the manufacturer.

### Statistical Analysis

Results are expressed as mean ± standard error of the mean mean (SEM). Differences between groups were assessed using the nonparametric Mann-Whitney U-test and p-values lower than 0.05 were considered as statistically significant. Analysis was performed using SPSS 18.0 (SPSS Inc., Chicago, IL, USA).

## Results

### Morphology and Immunophenotypic Profile

BM MSCs from all groups were expanded until the fifth passage and all displayed the characteristic spindle-shape morphology. Immunophenotypic assays at P2 and P4 did not identify any differences among groups. MSCs at diagnosis expressed CD90 (99.67±0.09%), CD105 (97.39±0.72%), CD146 (59.55±2.84%), CD29 (99.1±0.12%), CD44 (98.07±1.39%), CD95 (90.25±2.85%), and CD73 (99.4±0.4%), while there was no expression of hematopoietic markers such as CD34, CD45, and CD14. The same immunophenotypic profile was also observed at all treatment phases and in the control group.

### Growth Rate of MSCs (DT)

**Figure 1:**
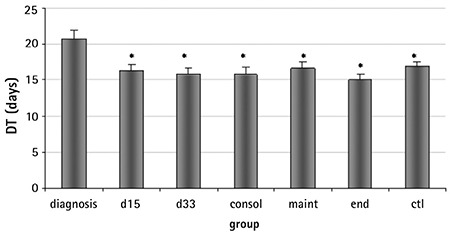
Days required for mesenchymal stromal cells in the mononuclear cells fraction (d0) to reach confluency. The doubling time at diagnosis differs from that of the phases of chemotherapy (p: d15=0.042, d33=0.007, consol=0.001, maint=0.022, end=0.002) and of the control (p=0.011). This defect subsides with the progression of culture (*: ss in comparison to the d group).

MSCs within the MNC fraction (d0) at diagnosis reached confluency in approximately 20.71±1.24 days, whereas at the end of chemotherapy they required 15.10±0.63 days. The DT at diagnosis was statistically different compared to all the phases of treatment (Figure 1). At subsequent passages, DT was similar among all groups (Table 1). This finding indicates that MSCs present in the MNC fraction at diagnosis, which was mainly constituted of lymphoblasts, expanded more slowly compared to treatment phases and the control group, but this defect subsided with the progression of culture (more advanced P). No difference was observed among all passages in all other studied groups. As the culture progressed, DT increased in all groups and the control.

### CFU-F Development

**Figure 2:**
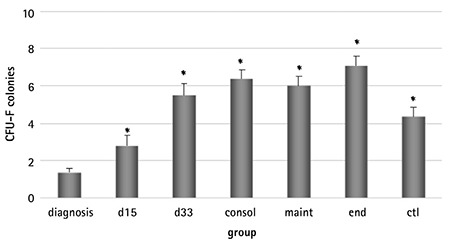
Colony forming units-fibroblast development of mesenchymal stromal cells in the mononuclear cells fraction (d0) from all studied groups. The number of colonies at diagnosis is lower than that of the other groups (d vs. end, control: p<0.0001). Culture progression resulted in lower colony development, becoming significant at the later passages. Data are expressed as mean ± SEM (*: p<0.05 compared to diagnosis). *CFU-F: Colony forming units.*

**Supplementary Figure 1:**
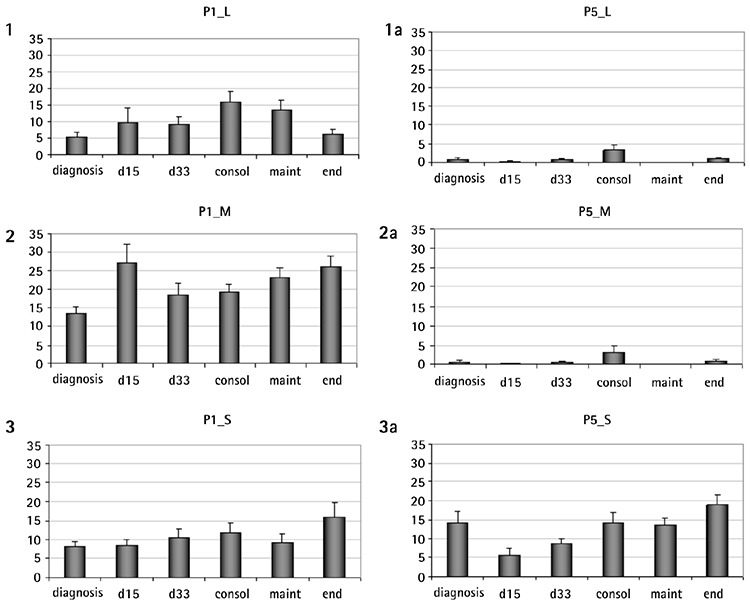
Colony forming units-fibroblast (CFU-F) colonies of large (L), medium (M), and small (S) size at the initial (P1) and last (P5) passages of the study. Larger colonies prevail at early passages while at the later ones the CFU-F population consists of mainly small colonies.

At day 0, the CFU-F formation at diagnosis appeared to be impaired compared to the other groups (Figure 2), a result attributed to the lower number of the medium and the large-sized colonies. The impaired clonogenicity of MSCs at the time of diagnosis was a constant finding, observed at subsequent passages as well (Table 2). Culture progression resulted in lower colony development, the control included, and this became statistically significant at the later passages (P1 vs. P4 or P5, p<0.001). MSCs at diagnosis formed fewer small, medium, and large colonies compared to all other groups. Larger colonies prevailed at early passages, while at the later ones, the CFU-F population consisted of mainly small colonies (Supplementary Figure 1).

### Cell-Cycle Analysis - Apoptosis

**Figure 3:**
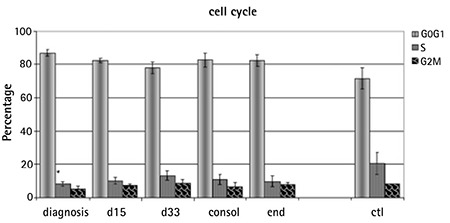
Analysis of the cell-cycle phases. Most of the mesenchymal stromal cells are in quiescence as the highest percentage of cells are in the G0G1 phase. Data are expressed as mean ± SEM.

Most of the MSCs were in quiescence, presenting a higher percentage of cells in the G0G1 phase compared to the control group (Figure 3). The study of apoptosis in all phases of disease and treatment at P2 and P4 confirmed the stability of BM-MSCs under long-term culture expansion through serial passages. Spontaneous apoptosis was detected at P2 and it did not change at P4 in all groups (Table 3).

### SDF-1α and Ang-1

SDF-1α in the MSC supernatants at diagnosis was variably expressed (median: 5334.63 pg/mL, range: 1066.70-22,480.86 pg/mL) and did not differ in comparison with the treatment phases. Its levels were higher in the HR group compared to the MR group (HR=9205.77±2721.82, MR=6686.11±4006.34, p=0.021).

**Figure 4:**
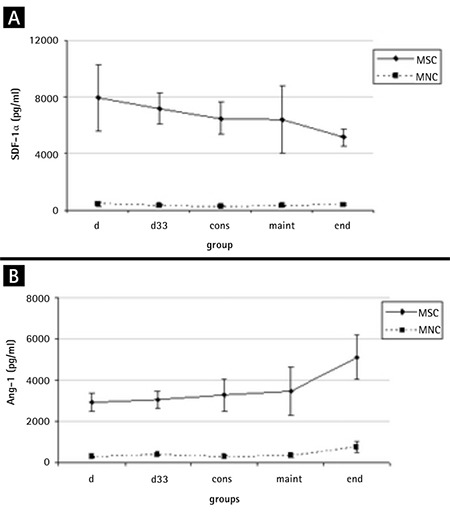
The stromal cell-derived factor-1α (SDF-1α) and angiopoietin-1 (Ang-1) expressions by both mesenchymal stromal cells (MSCs) and mononuclear cells (MNCs) at diagnosis and treatment. Stromal cells secrete higher amounts of both these factors. A) Variability in their expression was noticed at diagnosis, which became more uniform in treatment phases. B) No difference in angiopoietin-1 levels between diagnosis and treatment groups. *MSC: Mesenchymal stromal cell, MNC: mononuclear cell, Ang-1: angiopoietin-1, SDF-1α: stromal cell-derived factor-1α.*

As far as Ang-1 expression is concerned, in the two cell subpopulations of MNCs and MSCs, our results showed that, similar to SDF-1α, stromal cells secreted statistically significant higher amounts of this growth factor (Figure 4). No difference was found in the comparison of diagnosis with treatment groups.

## Discussion

MSCs are described as fibroblast-like cells, displaying a characteristic spindle shape, and all of our cells exhibited this feature. As in vitro culture progresses, cells enter senescence and MSCs become larger with irregular and flat shapes [26], not observed in our samples. Our source though was the BM of children, albeit leukemic BM, and our culture was followed up to P5 [27].

MSCs from all groups at different passages were highly expressing MSC-related markers and lacking the hematopoietic markers, as proposed by the International Society for Cell Therapy [28,29]. This indicates that the MSC cultures were homogeneous, in agreement with Conforti et al. [6], and neither disease nor treatment had any influence on them. Clonogenicity and proliferation potential were lower at diagnosis and decreased as the culture progressed, in partial agreement with the only study, so far, examining the characteristics of pediatric ALL-MSCs [6]. 

The lowest number of colonies was developed at diagnosis. Although this result does not stand alone to support that it is an intrinsic defect (because of the effect of the disease on MSCs) rather than a quantitative one, due to the lower frequency of MSCs in BM infiltrated by leukemic cells combined, with the fact that it continues to be seen in subsequent passages, where the same number of MSCs are used to initiate the culture, it is more suggestive of the hypothesis that the microenvironment (as expressed by BM MSCs) is also affected by the leukemic process. This result favors the observation of Conforti et al. [6] that leukemic cells do not confer to MSCs any preferential ability to proliferate, but they rather promote a deficient capacity, opposing the hypothesis that MSC populations might be crucial for the efficient promotion of the survival and proliferation of blasts [30]. Treatment does not affect the clonogenicity as the number of colonies produced at any time-point is similar to that of the controls. Another factor involved in colony development is the duration of the culture. Interestingly, the decrease of colony number throughout passages is more profound in large- and medium-sized colonies. Considering that large colonies derive from more primitive cells, it becomes obvious that older cultures contain more mature MSCs. Altogether, the above indicate that the presence of leukemia cells at diagnosis, but not chemotherapeutic agents, modifies BM-MSC properties.

Cell-cycle analysis revealed that most of the MSCs are in quiescence while about 20% of the cells of the control group are at the S phase, compared to less than 10% of the rest of the groups. Further analysis is required in order to fully clarify this difference found under identical culture conditions. Apoptosis remained unaltered throughout passages, a finding reported for BM-MSCs from children with benign hematological disorders [26]. Conforti et al. [6] reported different results, but they evaluated apoptosis for many passages and reported data for the latest one (P18).

Finally, we evaluated the levels of SDF-1α and Ang-1, recently revealed as major regulators in the crosstalk between hematopoietic progenitors and their microenvironment [31,32]. Data reporting the expression of SDF-1α by BM MSCs in patients with hematological malignancies are limited. SDF-1α in the supernatant of MSCs at diagnosis of ALL was slightly increased compared to that from treatment phases, although this difference was not statistically verified. Interestingly, HR patients exhibited higher levels compared to the MR ones, a difference no longer occurring upon treatment initiation. Reduced extracellular levels of SDF-1α were assessed in hematological malignancies of adults [33,34]. Others found increased SDF-1α secretion from MSCs at diagnosis in adolescents and young adults with ALL, reversed by chemotherapy [6]. In pediatric patients with acute leukemia, SDF-1α serum levels differed depending on whether they were evaluated in PB or BM serum (decreased expression) or MSC supernatants at diagnosis (decrease not evident) compared to the remission and control groups [15]. The above, combined with our findings, further support the notion that leukemic cells do not affect CXCL12 production and the decrease reported in serum cannot be attributed to the productive capacity of MSCs.

We found that the lowest amount of Ang-1 was expressed in MSC culture supernatant from diagnosis, albeit not statistically differently from treatment phases. There is one more study to date, on the effect of Ang-1 in childhood ALL [35], in which the authors claimed similar findings in the MSC supernatant and low levels of Ang-1 and Ang-2 in BM serum at diagnosis. Nevertheless, other factors such as age-related post-transcriptional effect on the expression of proteins or the exposure of BM MSCs to fetal bovine serum and FGF-b [36] have to be taken into consideration in order to fully exploit the role of these molecules in leukemia.

### Study Limitation

A limitation of our study is that the samples examined at different phases of ALL are not necessarily from the same patients longitudinally. This approach ensures a reasonable number of samples within a reasonable timeframe for each group for a rather rare pediatric entity and hence a stronger statistical result.

## Conclusion

In conclusion, biological characteristics and functional properties of MSCs are affected at the onset of leukemia. Most defects persist throughout passages. MSCs recover after treatment initiation and remission achievement and are not affected by chemotherapy. Their secretory profile remains unaltered by the disease. The summing of these data clearly indicates that any effect on MSCs from the leukemic clones in childhood ALL is transient and ceases upon treatment initiation. A standard hurdle in the comparison of our data to other studies continues to be the diversity of working protocols used for MSC cultures and further evaluation.

## Figures and Tables

**Table 1:**
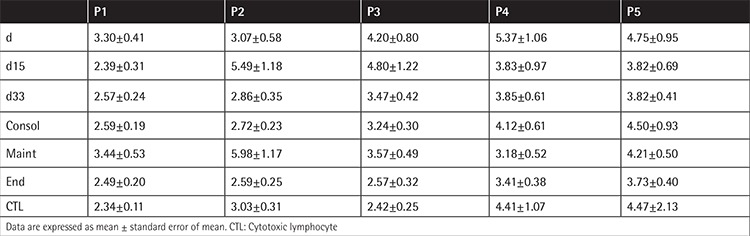
Doubling time of mesenchymal stromal cells of all groups in the different passages (P1-P5).

**Table 2:**
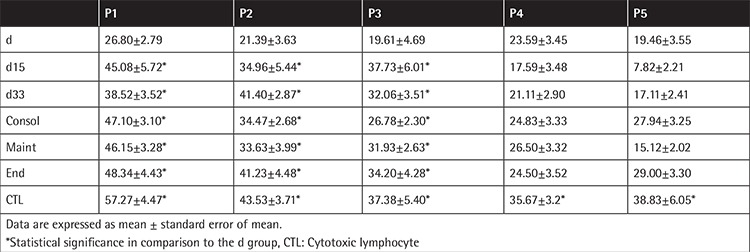
Colony forming units-fibroblast development of mesenchymal stromal cells from all studied groups (P1-P5).

**Table 3:**
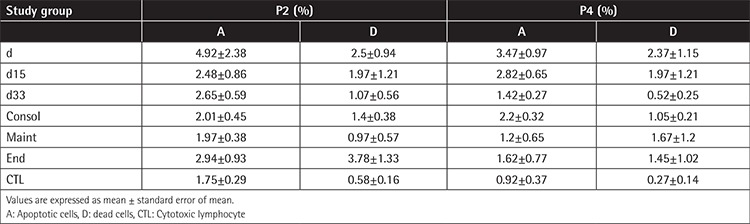
Spontaneous apoptosis, evaluated by flow cytometry after 7-amino-actinomycin D staining of mesenchymal stromal cells at diagnosis and during treatment at passages 2 and 4 (P2, P4).
