# Supplementary material for: Biological Features of Bone Marrow Mesenchymal Stromal Cells in Childhood Acute Lymphoblastic Leukemia
Source: Turk J Haematol. 2018 Mar 6;35(1):19–26. doi: 10.4274/tjh.2017.0209 (PMC5843770; doi:10.4274/tjh.2017.0209)
Supplement: Related texts

# Repy: Appendix: Supplementary Materials and Methods

## BM MNC Isolation and MSC Culture and Expansion

BM MNCs, following separation with Ficoll-Hypaque (1077 g/mL; Lymphoprep, Nycomed, Oslo, Norway), were cultured in a-MEM without nucleotides in the presence of 10% lot-selected fetal calf serum (Invitrogen Ltd., Paisley, UK) as described previously [21]. They were seeded at a concentration of 5x10^4^ cells/cm^2^ in the presence of 1 ng/mL FGF-2 (FGF-2; Abcys SA, Paris, France). A complete medium change was performed twice a week. When layers became confluent at ~90%, cells were detached using 0.25% trypsin/1 mM EDTA (Invitrogen Ltd.) and then replated at a concentration of 1x10^3^ cells/cm^2^ (passage 1, P1). MSCs were maintained in culture for up to five passages. Assays were performed at any of P1 to P4 depending on the cell availability.

## Immunophenotyping Evaluation

Phenotypic characterization of MSCs was performed by flow cytometry at various passages using the following monoclonal antibodies: CD105-phycoerythrin (PE) CD146-PE, CD73-PE CD29-fluorescein isothiocyanate (FITC), CD44-FITC, CD90-FITC, CD14-FITC, CD45-FITC, CD34-PE, and CD95-FITC (BD Biosciences, San Jose, CA, USA). One hundred thousand cells were stained with the markers as described previously [21]. At least 10,000 events were acquired for each analysis.

## Cell-Cycle Analysis - Apoptosis

MSCs, at either P2 or P3, after detachment by trypsinization (trypsin/EDTA 0.25%) were centrifuged at 150 x g for 10 min at 4 °C and washed with PBS. In order to estimate the percentage of cells in each phase of the cell cycle, 1x10^6^ MSCs were stained with 1 mL of propidium iodide staining solution (50 µg/mL propidium iodide, 1 mg/mL RNAse in PBS without Ca^++^/Mg^++^, pH 7.4) for 30 min at room temperature. After the acquisition of at least 10,000 events for each sample, cells were gated according to forward vs. side scatter (FSC/SSC) characteristics. Cell-cycle analysis was performed using WinMDI software, version 2.8 [22].

Apoptotic MSCs at passages P2 and P4 were detected by flow cytometry and 7-amino-actinomycin D (7-AAD; Sigma, St. Louis, MO, USA) staining [23]. They were initially gated according to their morphology (FSC/SSC). Then a scattergram was generated by combining FSC with 7-AAD fluorescence to quantitate 7-AAD^negative^ (alive), 7-AAD^low^ (early apoptotic), and 7-AAD^high^ (late apoptotic/dead) cells.

## Cell DT

DT was calculated according to the formula DT=*t*/n=*t*×log(2)/log (cells harvested/cells inoculated), where *t* is the time between initial plating and harvest for the respective passage.

## CFU-F Formation

At day 0.1x10^5^ MNCs were seeded in each well of a 24-well plate (in triplicate) in the absence of FGF-2. At subsequent passages, MSCs were plated in 20-cm^2^ petri plates at a concentration of 10 cells/cm^2^ (in duplicate). Following 14 days of culture at 37 °C and 5% CO_2_, CFU-F was quantified after staining with Giemsa stain and categorized according to size as small CFU-F (S: <50 cells), medium CFU-F (M: 50-500 cells), and large CFU-F (highly proliferating; L: >500 cells). The sum of CFU-F of all sizes is denoted as CFU-F.

## Detection of SDF-1α and Ang-1 (ELISA)

A quantitative sandwich ELISA was employed for the determination of both SDF-1α and Ang-1 in the supernatant of MSCs at any of P1 to P3 cultures (and of MNCs at d0) within the leukemia group only. All subgroups were examined for the evaluation of these factors through the whole course of the disease, diagnosis, and treatment. The ELISA kits were purchased from R&D Systems, and the instructions of the manufacturer were followed. More specifically, 100 µL for SDF-1α (50 µL for Ang-1) of standard or sample per well was added and incubated for 2 h at room temperature on a shaker. After well aspiration and washing, 200 µL of the corresponding conjugate was added. Incubation was continued for 2 h further under the same conditions. After washing, 200 µL of substrate solution was added to each well for 30 min at room temperature and then 50 µL of stop solution terminated the reaction. The optical density of each well was determined at 450 nm with wavelength correction at 570 nm.
